# Induction of Labour

**Published:** 1908-10-03

**Authors:** 


### OBSTETRICS.

### INDUCTION OF LABOUR

When induction of premature labour has been decided upon for contracted pelvis in the manner described in our previous article, certain definite steps in technique are necessary. Of the many ways of inducing labour, that by the bougie is the only one in common use in cases of pelvic contraction. The bougie is of gum elastic and about the size of a No. 10 catheter. It is hollow but has no eye, and the end is closed by an ivory button. It is best sterilised by boiling, and a convenient way is to place it first in a wide glass tube having indiarubber corks at each end. The corks must be taken out and the tube, bougie and corks boiled together. The corks may then be replaced and the bougie can be carried sterile to the patient.

Passing the bougie into the uterus is not an easy operation, and must be performed in such a manner as to introduce no septic material and not to rupture the membranes. Before passing the bougie the vagina must be rendered aseptic by douching with an antiseptic, or better by swabbing out with pledgets of cotton-wool on sterile forceps. Lysol ʒj. ad Oj. is perhaps the best antiseptic for this purpose. The bougie should be passed by the sense of sight and not by touch alone.

For this purpose a speculum to draw back the perineum should be made use of, and a vulsellum should be inserted into the anterior lip of the os uteri so as to steady it. The freshly-boiled bougie is soft, and on this account does not always pass readily; its passage may be facilitated by cutting off the ivory-capped end and stiffening it with a catheter stilet. A wide curve may be imparted to the bougie with the stilet in, so that it may the more readily pass round the internal os and up the anterior wall of the uterus. Any obstruction should be a signal for slight withdrawal and passage in another direction. The stilet should be withdrawn when about five inches of the bougie have been introduced. The bougie is then to be pushed on until about an inch sticks out of the cervix. The vagina is then lightly plugged with sterile gauze and the patient left.

The patient need not be confined to bed, but may be allowed to sit about her room and even get about the house. Labour sometimes does not come on for many hours, and occasionally it is necessary to introduce more than one bougie. The onset of labour pains generally drives out the bougie, which is found curled up in the vagina.

In conducting an induced labour it is very important that the membranes should remain unruptured, for it is only by their aid that the os uteri will be opened up in normal time. If the membranes rupture prematurely the first stage will be unduly prolonged, because in many instances the fœtal head does not descend easily enough to dilate the cervix in lieu of the bag of waters. If the first stage is unduly prolonged for this reason the fœtus sustains prolonged pressure, and consequently the chances of its survival become smaller. It is recommended in such a case to dilate the cervix artificially with a de Ribes' bag, provided this can be done without introducing sepsis and displacing the presenting part. It must not be forgotten, however, that interference is the thing to avoid in induced labour; the more normally labour proceeds after induction the more likely the child is to survive.

When the fœtal head enters the brim spontaneously the second stage should be allowed to proceed as long as possible without interference, consonant with the safety of the mother. Premature infants will not stand much instrumental violence, but are not injured by a little prolongation of a natural second stage.

Further, it must be remembered that the passages are not so easily dilated before the normal end of the pregnancy, because they have not the same degree of physiological relaxation. Hence a longer second stage may be necessary on this account. The guide to the moment for interference is *the mother's pulse*, too often neglected in the conduct of labours, normal or abnormal. While the pulse remains below 100 the case may be left alone as long as there is any indication that the fœtus is advancing.

The fœtal pulse must sometimes be taken into account; rapid delivery is indicated if the fœtal heart beat falls below 100 or rises above 160. But no operation must be undertaken for rapid delivery unless this can be accomplished without danger to the mother. Any operation which risks an injury to the mother is almost certain to mean death to the fœtus on account of the pressure necessary to effect delivery. If, however, the fœtal head does not enter the brim spontaneously the forceps must be applied above the brim, a procedure very seldom required and hardly likely to be followed by the birth of a living child. Axis traction forceps are of great service in a case of this kind, but care must be used lest too much pressure is put upon the fœtal head for too long at a time, and the lock of the forceps should be frequently undone on this account. The aim should be to deliver slowly.

In most hands vertex delivery is safer than breech, but it sometimes happens that the child presents by the breech and must then be delivered in this position. The safeguard here is to leave the case alone absolutely until the chest is born, and then to release the arms as quickly as possible and prevent the head from extending.

The fœtal head will enter the brim base first fairly easily, but is liable to extend, and this, together with the probable extension of the arms leads to just enough delay to be fatal. If the child dies before delivery of the head, perforation of the aftercoming head should be performed, so as to deliver with the least risk of injury to the mother. This is always an easy operation in minor degrees of contraction, and as a rule is best performed through the occipital bone.

